# Predicting Weaning Weight of Romanov Lambs From Biometric Measurements Before Weaning Age Using Machine Learning Algorithms

**DOI:** 10.1002/vms3.70420

**Published:** 2025-05-28

**Authors:** Mehmet Eroğlu, Ali Osman Turgut, Mürsel Küçük, Muhammed Furkan Önen

**Affiliations:** ^1^ Department of Animal Science Faculty of Veterinary Medicine, Siirt University Siirt Türkiye

**Keywords:** gradient boosting, machine learning, predict, weaning weight, XGBoost

## Abstract

**Background:**

Machine learning systems learn from historical data to forecast future outcomes. In the context of livestock farming, machine learning can be utilized to predict variables such as growth rates, milk production and breeding success by analysing data related to animal health, nutrition and environmental conditions.

**Objective:**

This study aimed to investigate the performance of different machine learning algorithms in predicting weaning weight based on biometric measurements of Romanov lambs at 30 days of age.

**Methods:**

The biometric traits of the lambs, including body length (BL), chest circumference (CC), chest depth (CD), chest width (CH), withers height (WH), rump height (RH), rump width (RW) and sex were used to construct predictive models. The study employed random forest (RF), classification and regression trees (CART), gradient boosting (GB), eXtreme gradient boosting (XGBoost) and CatBoost algorithms. The data was standardized to eliminate scale differences and divided into training (80%) and test (20%) sets. GridSearchCV was utilized for hyperparameter optimization. The performance of the models was evaluated using various goodness‐of‐fit metrics, including RMSE, MAE, *R* ^2^, MAPE, RAE, MAD and SD ratio.

**Results:**

The gradient boosting and XGBoost models performed the highest *R* ^2^ values and the lowest RMSE, MAE and MAPE values in the test data. In contrast, the random forest and CatBoost models showed lower predictive performance, with higher errors in the test data.

**Conclusion:**

The study suggests that machine learning algorithms, particularly gradient boosting and XGBoost, show promising potential in predicting the weaning weight of lambs. These insights may facilitate more informed decision‐making in animal breeding and selection, potentially contributing to enhanced livestock management practices.

## Graphical abstract

This study assessed various machine learning algorithms for predicting weaning weight in Romanov lambs using biometric traits. Gradient Boosting and XGBoost outperformed other models, showing the highest *R* ^2^ and lowest error metrics (RMSE, MAE, MAPE). These findings suggest that machine learning can significantly improve breeding practices by providing accurate weight predictions for better management of livestock.

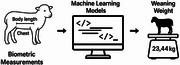

## 1 Introduction

Weaning age varies based on the production goals and the characteristics of the lambs, such as age, weight and their ability to consume solid food. Weaning is a critical aspect of managing ewes and lambs. In traditional sheep production systems, lambs are suckled by their mothers and are typically weaned at 60–90 days of age (Freitas‐de‐Melo et al. 2022). Because weaning may affect the lamb's health in addition to the relationship between the ewe and the lamb, early weaning causes immunological, physiological and behavioural changes that harm the health of both ewes and lambs. It also presents concerns related to lamb growth and animal welfare (Napolitano et al. 2008). Thus, keeping an eye on the weight of the lamb during its growth is essential for managing its health and guaranteeing the effectiveness of the manufacturing process. However, according to Santos et al. (2020), there are significant obstacles because many producers lack the infrastructure or technologies required for such monitoring, which makes it rare. As a result, simple procedures like biometric measurements can be used to solve performance concerns and enable weight monitoring, making them an effective tool for estimating animal weight. There has been extensive research in the literature on predicting the body weight of farm animals based on different body features seen at different growth periods for cattle, sheep and goats (Ozkaya and Bozkurt 2009; Atta et al. 2024).

Artificial intelligence (AI) and machine learning (ML) are increasingly transforming agriculture, livestock management and veterinary education (Choudhary et al. 2023). In sustainable farming, these technologies are optimizing livestock management by analysing large datasets, including medical histories, laboratory results and imaging scans, to enhance diagnostic accuracy. AI‐driven systems identify patterns, detect subtle irregularities and apply geometric morphometric analysis to assess shape differences, while also optimizing feeding strategies, detecting early signs of disease and monitoring growth performance (Choudhary et al. 2024; Vickram et al. 2024; Gundemir et al. 2024).

Moreover, advancements in data mining and ML have enabled predictions of live body weight based on morphological characteristics, paving the way for precision farming and sustainable livestock management (Ağaoğlu and Gürcan 2023). Specifically, in sheep management, live weight is frequently predicted using several ML algorithms, including random forest (RF) (Huma and Iqbal 2019; Tırınk, 2022), classification and regression trees (CART) (Ali et al. 2015; Olfaz et al. 2019; Faraz et al. 2023) and eXtreme gradient boosting (XGBoost) or gradient boosting (GBoost) (Faraz et al. 2023).

The ML approach was effectively applied to the live weight prediction of 93 Norduz sheep with the use of seven morphological measurements. Comparing the predictive capabilities of SVMR, CART, RF and MANN models, the author found that RF performed better than MANN, SVMR and CART models with the lowest goodness of fit criteria values (Çakmakçı 2022). Two ML algorithms, XGBoost and MARS, were recently used by Faraz et al. (2023) to forecast the body weight of 152 Kajli sheep. Height at wither, body length, heart circumference, head length, head width, rump length and rump width were among the physical measurements recorded in the study. The XGBoost algorithm produced the best results. Similarly, Tırınk et al. (2023) found that RF performed better than the other models when estimating body weight in Suffolk × Polish Merino sheep based on seven body measurements, with chest circumference as the primary predictor.

This study evaluated the performance of various ML models for predicting the weaning weight (60 days of age) of Romanov lambs based on biometric measurements at 30 days of age, with the aim of contributing to more efficient and economic decisions in animal breeding and selection programs.

## 2 Materials and Methods

This study used 49 Romanov lambs ranging from 3 to 6 years of maternal age at the Small and Large Animal Breeding, Practice, and Research Center, Siirt University. The lambs' biometric measurements of body length (BL), chest circumference (CC), chest depth (CD), chest width (CW), withers height (WH), rump height (RH) and rump width (RW) were taken at 30 days of age. These data and sex information were used for training ML models and performance evaluations for predicting the weaning weight (60 days of age).

In this study, RF and CART were utilized as part of the decision tree‐based methods, while GB, XGBoost and CatBoost algorithms were employed as examples of ensemble methods. In order to eliminate the scale differences of the features in the data set, the data set was standardized with the standard scaler method. This process ensured that the mean of each feature was normalized to zero and the standard deviation as one. The data set was divided into training (80%) and testing (20%). The training set is used in the model's learning process, while the test set is reserved for evaluating the model's accuracy and generalization performance. GridSearchCV (10‐fold cross‐validation) was used to select the parameter sets that give the best results in the specified hyperparameter ranges. This method was preferred to enable the models to make predictions with higher accuracy rates.

### 2.1 Random Forests

RF are machine‐learning tools that create multiple decision trees during training. Each tree is constructed by selecting random samples of the data and making splits based on input features that minimize the mean squares error for regression (Hay 2024). RFs are popular among multivariate statistical methods due to their easy applicability in classification and regression‐type problems (Tırınk 2022).

### 2.2 Classification and Regression Tree (CART)

Breiman et al. (1964) first presented the CART procedure. The binary split tree structure that contains the two sub‐nodes is produced when a variable is split homogeneously by the CART algorithm. The CART technique begins with the root node and the original data set and continues until several homogeneous subnodes are obtained, providing the least error variation.

### 2.3 Gradient Boosting

Boosting algorithms are techniques that gradually merge learners, each slightly better than random learners, to form more robust models. One example is GBoost, which is based on decision trees and operates similarly to the RF algorithm. Moreover, GBoost can be classified as an ensemble method (Jun 2021). Its distinct approach to ensemble construction sets it apart from other algorithms. The algorithm sequentially integrates different explanatory variables, applying partial shrinkage to each, making it helpful in selecting relevant variables. In contrast to RF, GBoost involves sequentially adding trees to the ensemble, with each tree adjusted based on the cumulative error of the ensemble's predictions (Freeman et al. 2016)

### 2.4 XGBoost

XGBoost is a ML algorithm based on decision trees that employs a gradient‐boosting framework. It applies to both regression and classification tasks. The XGBoost model is initially estimates by learning from a tree constructed from the sample data. Then it builds a second tree to account for the difference between the predicted and actual outcomes. This process allows XGBoost to correct errors from the previous decision trees and adjusts the objective function to mitigate overfitting. Key hyperparameters for constructing the XGBoost model include the maximum tree depth, learning rate and the number of iterations (Chen and Guestrin 2016).

### 2.5 CatBoost

A key component of the CatBoost algorithm, a machine‐learning technique based on gradient‐boosted decision trees, is the creative and effective handling of categorical features. It also reduces gradient bias and prediction deviation, which improves the algorithm's accuracy and capacity for generalization. Divergent from the usual gradient boosted decision trees algorithm, CatBoost combines methods of random permutation and mean label value calculation for sample processing, in addition to including a prior distribution term, successfully minimizing the noise effects from low‐frequency categorical data. CatBoost uses a perfectly symmetric tree as the foundation model to further optimize processing capacity for high‐dimensional sparse data (Hancock and Khoshgoftaar 2020).

A comprehensive set of goodness‐of‐fit metrics was used to assess the performance of the predictive models in estimating weaning weight. These metrics included coefficient of determination (*R* ^2^), root mean square error (RMSE), mean absolute error (MAE), mean absolute percentage error (MAPE), relative absolute error (RAE), mean absolute deviation (MAD) and standard deviation ratio (SD ratio) (Tırınk et al. 2023). Specifically, RMSE, MAE, *R* ^2^, MAPE, RAE, MAD and SD Ratio were calculated for both training and test data sets to assess the accuracy and generalizability of the models. The optimal model was selected based on criteria such as the lowest RMSE, MAE, MAPE, RAE, MAD and SD values and the highest *R* ^2^ values in both training and test datasets for predicting weaning weight in Romanow lambs (Tatliyer 2020).

1. Root mean squared error (RMSE)
$$\mathrm{RMSE}\;=\sqrt{\frac{1}{n}\sum_{i=1}^{n}{\left(y_{i}-{\hat{y}}_{i}\right)}^{2}}$$
2. Mean absolute error (MAE)
$$\mathrm{MAE}\;=\frac{1}{n}\;\sum_{i=1}^{n}|y_{i}-{\hat{y}}_{i}|$$
3. Coefficient of determination (*R* ^2^)
$$\;R^{2}=1-\frac{\sum_{i=1}^{n}{\left(y_{i}-{\hat{y}}_{i}\right)}^{2}}{\sum_{i=1}^{n}{\left(y_{i}-\hat{y}\right)}^{2}}$$
4. Mean absolute percentage error (MAPE)
$$\mathrm{MAPE}\;=\frac{1}{n}\;\sum_{i=1}^{n}|\frac{y_{i}-{\hat{y}}_{i}}{y_{i}}|\times 100$$
5. Relative absolute error (RAE)
$$\mathrm{RAE}\;=\frac{\sum_{i=1}^{n}|y_{i}-{\hat{y}}_{i}|}{\sum_{i=1}^{n}|y_{i}-\hat{y}|}$$
6. Mean absolute deviation (MAD)
$$\mathrm{MAD}\;=\frac{1}{n}\;\sum_{i=1}^{n}|y_{i}-\hat{y}|$$
7. Standard deviation ratio (SD Ratio):
$$\mathrm{SD}\;\mathrm{Ratio}\;=\frac{\sqrt{\frac{1}{n-1}\sum_{i=1}^{n}{\left(y_{i}-\hat{y}\right)}^{2}}}{\sqrt{\frac{1}{n-1}\sum_{i=1}^{n}{\left(y_{i}-\bar{y}\right)}^{2}}}$$

Where *n* is the number of observations, *y_i_* is the actual value, $\left(\hat{y}\right)$ is the predicted value, *y* is the mean of actual values.

## 3 Results

Table 1 presents descriptive statistics of different physical traits at 30 days of age, separated by the Romanow lambs' sex. While the number of observations (*n*) for female lambs is 28, this number is 21 for males. According to Table 1, the average weaning weight was 10.74 and 11.98 kg in females and males, respectively. All measures (WW, BL, CC, WH, RH, RW, CW and CD) show slightly higher means in males. These findings highlight differences and variations in physical characteristics between sexes, which should be considered when developing weaning weight prediction models.

**TABLE 1:** Descriptive statistics of biometric measurements by sex at 30 days of age.

	*n*	mean	std	min	25%	50%	75%	max
FEMALE								
WW (kg)	28	10.74	2.87	5.30	9.84	10.79	12.39	16.14
BL (cm)	28	39.39	4.52	28.87	37.25	39.33	41.89	49.75
CC (cm)	28	47.84	5.17	33.00	45.22	48.12	51.78	55.42
WH (cm)	28	42.31	3.50	32.60	40.66	42.80	44.06	49.00
RH (cm)	28	42.19	3.45	32.60	40.62	42.88	44.83	47.42
RW (cm)	28	12.63	1.59	9.81	11.77	12.27	13.37	16.57
CW (cm)	28	12.56	1.63	9.42	11.38	12.36	13.64	16.50
CD (cm)	28	19.55	2.75	14.30	18.30	19.45	21.10	25.42
MALE								
WW (kg)	21	11.98	2.44	5.34	10.69	12.08	13.28	16.08
BL (cm)	21	40.38	3.30	35.97	37.42	40.10	42.16	48.41
CC (cm)	21	49.52	3.81	40.78	47.30	49.75	51.57	55.88
WH (cm)	21	42.73	3.39	37.28	40.25	42.50	46.23	48.20
RH (cm)	21	42.79	3.46	36.85	40.25	42.81	46.23	48.00
RW (cm)	21	13.02	1.31	10.66	11.85	12.81	13.64	15.50
CW (cm)	21	13.06	1.32	11.33	11.85	12.81	14.00	15.75
CD (cm)	21	20.13	3.52	13.30	18.00	20.50	22.83	25.38

Abbreviations: BL, body length; CC, chest circumference; CD, chest depth; CH, chest width; RH, rump height; RW, rump width; std, standard deviation; WH, withers height.

Table 2 shows the correlation between weaning weight and morphological traits in 30‐day‐old lambs. These results show that weaning weight is mainly influenced by CC and RH, while sex has the least effect.

**TABLE 2:** Correlation matrix between morphological traits at 30 days of age and weaning weight.

	WW	BL	CC	WH	RH	RW	CW	CD	SEX
**WW**	1.00								
**BL**	0.57	1.00							
**CC**	0.65	0.42	1.00						
**WH**	0.57	0.65	0.52	1.00					
**RH**	0.60	0.63	0.54	0.97	1.00				
**RW**	0.45	0.56	0.18	0.42	0.40	1.00			
**CW**	0.44	0.51	0.24	0.37	0.36	0.96	1.00		
**CD**	0.51	0.47	0.52	0.74	0.71	0.14	0.10	1.00	
**SEX**	0.23	0.12	0.14	0.01	0.04	0.13	0.17	0.06	1.00

Abbreviations: BL, body length; CC, chest circumference; CD, chest depth; CH, chest width; RH, rump height; RW, rump width; WH, withers height; WW, weaning Weight.

**FIGURE 1:**
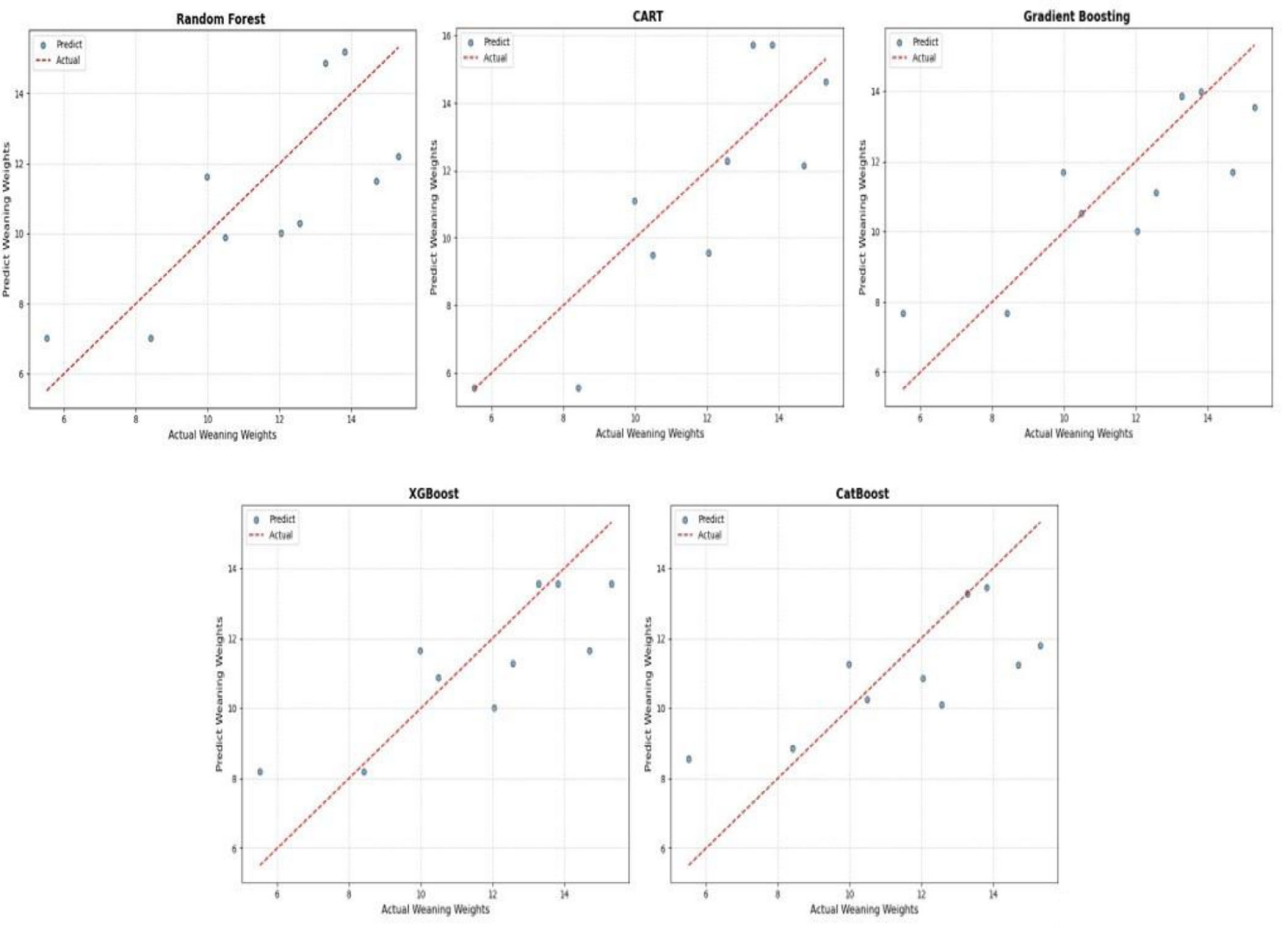
Distribution of Model Predictions and Actual Values, Classification and Regression Trees (CART), Extreme Gradient Boosting (XGBoost).

Table 3 compares all algorithms and goodness of fit criteria. For all algorithms, the performances of the training and test sets were evaluated. For each model, the performance values obtained from the test set were weaker than those obtained from the training data set. The RF and CART models had the lowest RMSE (0.92 and 0.74, respectively) and MAE values on the training data. However, in the evaluations of the test data, RF (2.01) and CATBoost (2.07) show the highest RMSE values. The *R* ^2^ values show that the RF (0.88) and CART (0.92) models have strong predictive power in the training set, while these models underperform in the test set. The distributions of the actual and predicted test set values are shown in Figure 1. RF (16.47) showed the highest MAPE on the test data, while CATBoost (11.75) performed the worst on the training set. The RAE and MAD metrics similarly show that RF and CATBoost models perform better on the training data. However, on the test data, RF (0.77) has the highest RAE value, and the test MAD value (1.86) is higher than the other models. MAD represents the deviation rate of the predicted values from the actual values. In the train data, the MAD value of the CART model is 0.58 and has the lowest rate. In the test data, the lowest MAD value belongs to the XGBoost model with 1.36. The MAD values of GBoost, CART, CatBoost, and RF models were calculated as 1.37, 1.54, 1.60 and 1.87, respectively. SD ratio evaluates the model's prediction performance. The lowest SD Ratio value in the train data belongs to the XGBoost model with 0.52. The lowest SD Ratio value in the test data belongs to the CatBoost model, which has 0.53.

**TABLE 3:** Performance metrics for models.

	RF	CART	Gradient boosting	XGBoost	CatBoost
**Train RMSE**	0.93	0.74	1.19	1.36	1.41
**Test RMSE**	2.02	1.82	1.64	1.69	2.07
**Train MAE**	0.69	0.58	0.91	1.01	1.07
**Test MAE**	1.87	1.54	1.37	1.36	1.60
**Train *R* ^2^**	0.88	0.92	0.80	0.74	0.73
**Test *R* ^2^**	0.51	0.60	0.68	0.66	0.48
**Train MAPE**	7.59	5.47	10.01	11.23	11.76
**Test MAPE**	16.47	13.23	13.16	13.47	15.40
**Train RAE**	0.34	0.28	0.45	0.50	0.53
**Test RAE**	0.78	0.64	0.57	0.56	0.66
**Train MAD**	0.69	0.58	0.91	1.01	1.07
**Test MAD**	1.87	1.54	1.37	1.36	1.60
**Train SD Ratio**	0.79	0.96	0.59	0.52	0.53
**Test SD Ratio**	0.91	1.22	0.75	0.66	0.53

Abbreviations: CART, classification and regression trees; CC, chest circumference; CD, chest depth; CH, chest width; MAD, mean absolute deviation; MAE, mean absolute error; MAPE, mean absolute percentage error; *R* ^2^, coefficient of determination; RAE, relative absolute error; RF, random forest; RH, rump height; RMSE, root mean square error; RW, rump width; SD ratio, standard deviation ratio; WH, withers height; XGBoost, extreme gradient boosting.

**FIGURE 2:**
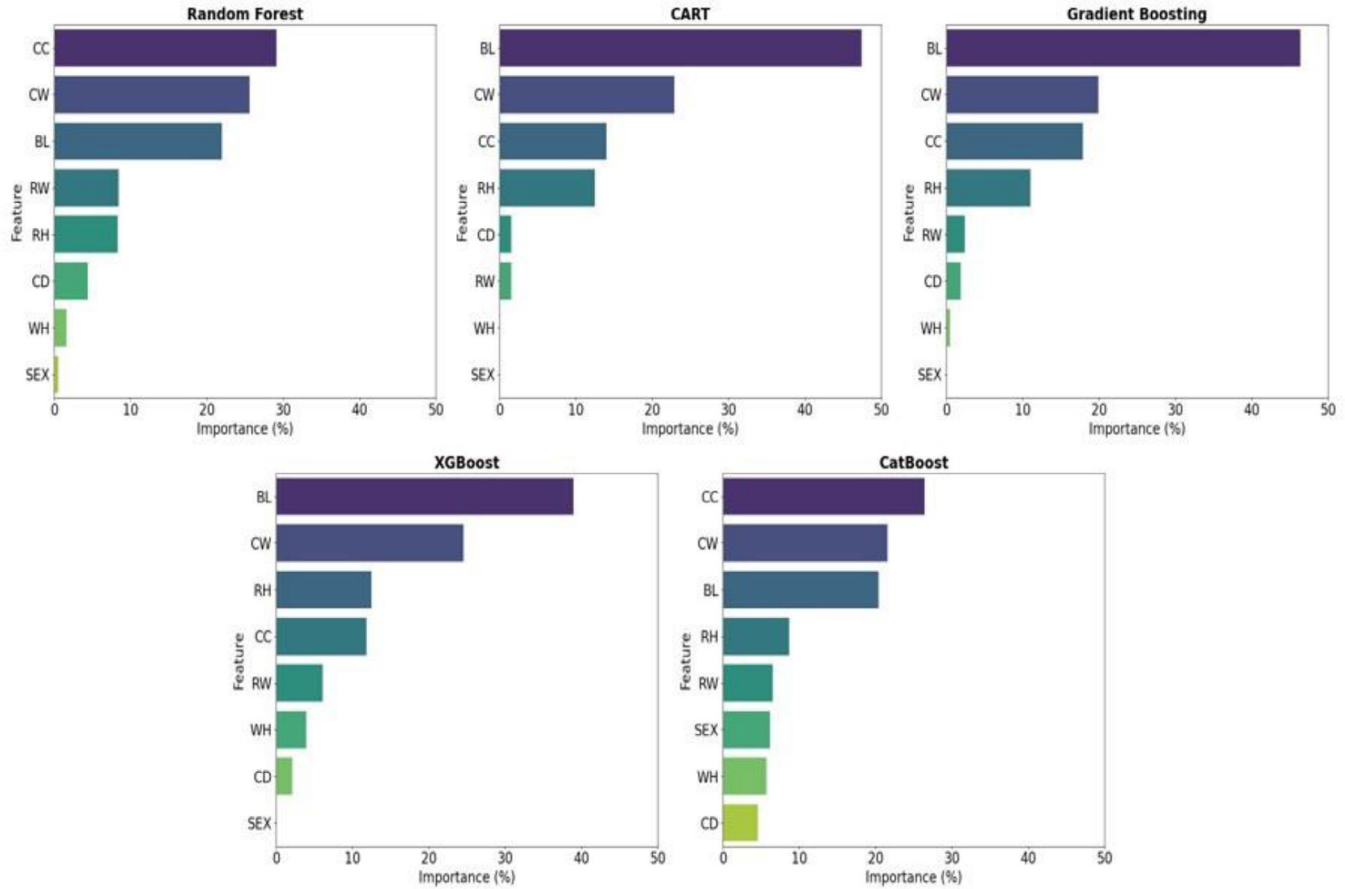
Feature importance scores for the models, RF: Random Forest; CART: Classification and Regression TreesXGBoost: Body length; CC: Chest circumference; CD: Chest depth; CH: Chest width; WH: Withers height; RH: Rump height; RW: Rump width.

Figure 2 shows the importance of biometric features in the models. BL was the most important parameter in the CART, XGBoost and GBoost models, which performed better in the test sets. Similarly, sex did not show any significance in these three models. CC and CW factors showed more importance in the lower‐performing RF and CatBoost models.

**FIGURE 3:**
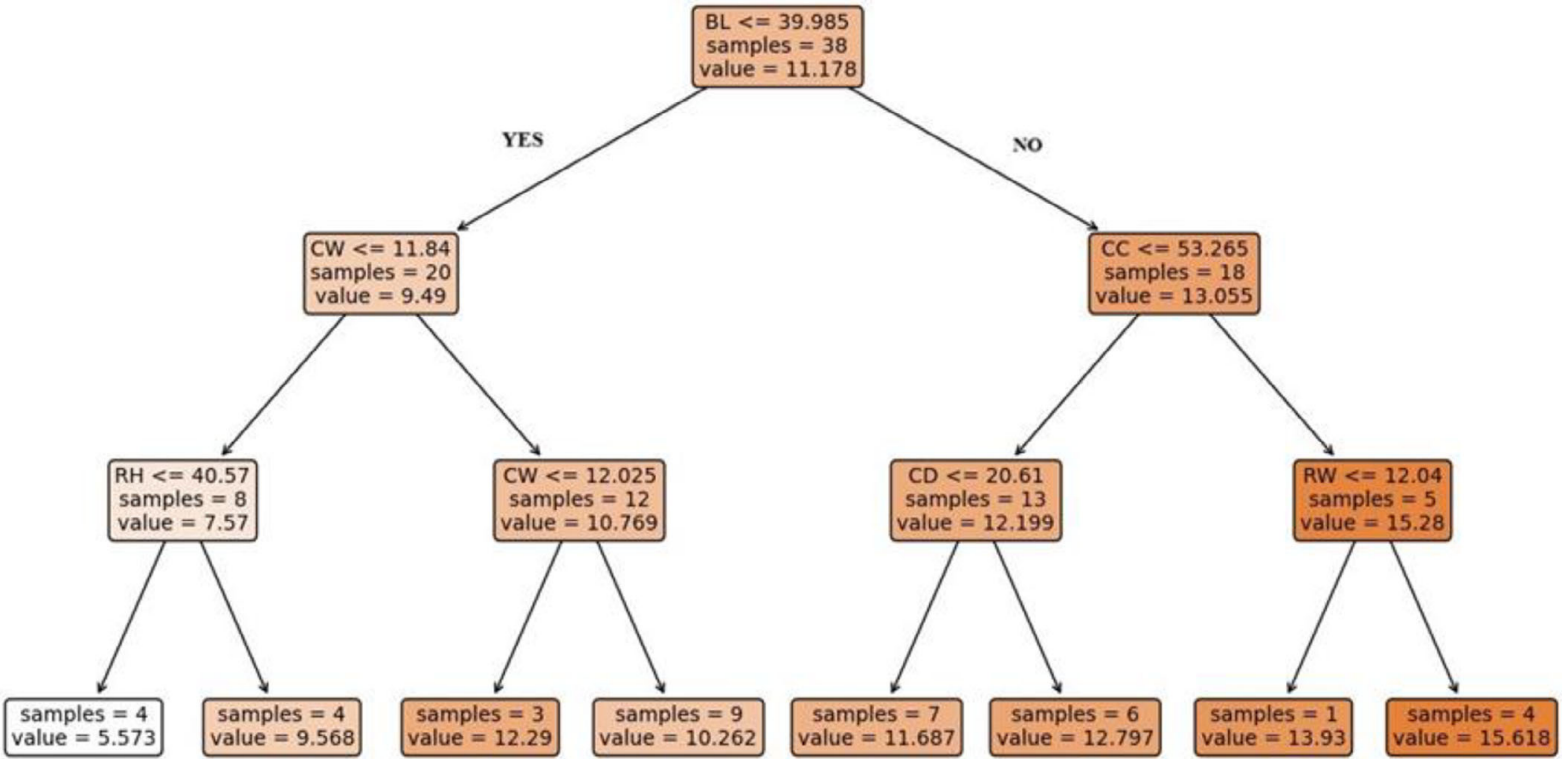
Regression tree diagram of the Classification and Regression Trees (CART) model to predict weaning weight, CC: Chest circumference; CD: Chest depth; CH: Chest width; WH: Withers height; RH: Rump height; RW: Rump width.

As illustrated in Figure 3, the CART model's regression tree is utilized to predict weaning weight. Initially, the model comprised 38 root node samples, subdivided based on the BL feature. The mean prediction value for this group was determined to be 11.178. The data with a BL value less than 39.985 are divided into left branches, and those with a BL value greater than 39.985 are divided into right branches. There are 20 samples in the left branch, and this group's average prediction value was 9.49. There are 18 samples in the right branch, and the average prediction value was 13.055. This initial division underscores the significance of the BL30 feature in the model. As the tree becomes more complex, the node is subdivided into an increasing number of subnodes. The data on the left branch was further categorized into two subgroups based on the rule CW ≤ 11.84. This subdivision facilitates more precise predictions by the model. As the leaf nodes were reached, the groups were divided into smaller subsets and the number of samples within the group and the prediction value were calculated for each leaf node. For example, while the predicted value for four samples in one leaf node was 5573, it was 15,618 in another. In particular, features such as ‘BL’ and ‘CW’ are important variables that increase the model's accuracy.

## 4 Discussion

ML models are data‐driven learning algorithms that can learn from historical data and predict future scenarios, such as weaning weight, which is the focus of this study. The success of these models depends on the selection of appropriate algorithms and the evaluation of these models using suitable performance metrics. In this study, the GBoost, XGBoost and CART algorithms demonstrated the highest *R* ^2^ values and the lowest RMSE, MAE, MAPE, RAE and MAD values in the test data. Thus, the models perform better than the RF and CatBoost models. The GBoost Model has produced an acceptable generalization with a 13.16 MAPE value, 1.64 RMSE value, 1.37 MAE value and 0.68 *R* ^2^ value in the test set. Furthermore, the relatively minor discrepancy between the training and test data suggests that this model outperforms alternative algorithms. The XGBoost model obtained 0.65, 1.69 and 13.47 results from the *R* ^2^, RMSE and MAPE criteria. Although these values are close to those obtained by GBoost, XGBoost performs slightly less well. This finding indicates that while the overall accuracy of XGBoost is satisfactory, its generalization ability is marginally inferior to that of GBoost.

Olfaz et al. (2019) used CART and CHAID data mining algorithms to investigate the effect of sex, birth type, farm type, birth weight and weighing time on weaning weight in Karayaka sheep. Similar to this study, the CART algorithm showed lower performance. Therefore, they suggested using the CHAID algorithm, which is biologically more useful than CART. Although no studies in the literature specifically use ML algorithms to predict weaning weight before reaching weaning age with the help of biometric measurements, many studies have been conducted to predict body weight.

The results of this study are consistent with the results reported by Hamadani and Ganai (2023), who, in their study on body weight prediction, also identified the GBoost and XGBoost algorithms as high‐performance models. In addition, Niang et al. (2021) demonstrated that the XGBoost algorithm attains a reduced error value compared to RFs. The superiority of XGBoost in terms of model generalization capabilities may be attributed to its utilization of advanced regularisation (L1 and L2). The CART model demonstrated effective generalization, as evidenced by the *R* ^2^ value of 0.60 in the test and other metrics, with a marginal discrepancy between the training and test metrics. This finding suggests that the model exhibits balanced performance across training and test data. The CatBoost model demonstrated the poorest performance on the test data. The test RMSE was 2.07, and the test MAE was 1.60, higher than the other models. The test *R* ^2^ value is 0.48, and the test MAPE value is 15.40, indicating the highest error. This finding suggests that the model tends to make more errors when generalizing in the test data and that the accuracy of the predictions is low for weaning weight.

In their study, Huma and Iqbal (2019) aimed to predict the body weight of 131 male Balochi sheep by employing various body and testicular measurements in ML models. The study concluded that, in contrast to the present study's findings, the RFs model exhibited the optimal performance for body weight estimation and provided more precise predictions by attaining superior outcomes compared to traditional linear models. Consequently, they proposed that the RFs method can be highly effective for modelling and predicting body weight through various biometric and testicular traits in small ruminants. Canaza‐Cayo et al. (2024) utilized various ML models, including CART, XGBoost and RF, to predict body weight. Similar to this study, it was determined that BL emerged as a significant trait in predicting body weight. However, in contrast to the present study, the RF model emerged as the most effective model, achieving the highest *R* ^2^ value and the lowest MAE, RMSE and MAPE values. However, the XGBoost algorithm similarly demonstrated superior performance compared to the SVMR, CART, MANN and MARS algorithms.

Vázquez‐Martínez et al. (2023) operated multivariate adaptive regression splines (MARS), CART and support vector machine regression (SVR) algorithms to verify body weight in 280 hair sheep. The researchers reported that the strongest correlation with body weight was observed for heart circumference (0.97), while the weakest correlations were found for BL and body height (0.73 and 0.83, respectively). Subsequently, the SVR algorithm was identified as the most appropriate for both the training and test sets, as it exhibited superior performance metrics, including an *R* ^2^ value of 0.94. It demonstrated the test sets' lowest RMSE, SD ratio, CV and MAPE values. Tırınk et al. (2023) stated that the RF model was the most appropriate algorithm for predicting body weight from various biometric measurements in Suffolk and Polish Merino genotype sheep. This conclusion was based on the fact that the model gave close results for both the training and test sets and gave the highest *R* ^2^ and the lowest RMSE, SD ratio, CV and MAPE values in the test set.

In their study, Kozaklı et al. (2024) used a range of algorithms, including RF, support vector machines (SVM), SVR, XGBoost and GBoost, to predict the post‐weaning weights of Akkaraman lambs reared in different farms. The ensuing comparison results indicated that the RF algorithm performed better than the other algorithms, with an Adj‐*R* ^2^ of 0.75, RMSE of 3.683, MAD of 2.876 and MAPE of 10.112. This outcome is inconsistent with the results of this study. Faraz et al. (2023) aimed to use the XGBoost and MARS algorithms to predict body weight from bimetric measurements. Similar to this study's results, they recommended the XGBoost algorithm as a reliable model. This model could be feasible in rural areas where traditional scales may be inaccessible or unreliable.

Tırınk et al. (2022) conducted a comparative study using BRNN, SVR, RFR and MARS algorithms to predict body weight from biometric measurements (BL, CC, ear length, ear width, head width, head length, WH, rump length, RW, neck length and neck width) for the Thalli sheep breed. The findings of this study indicated that the MARS algorithm exhibited superior performance compared to the neural network, RF regression and support vector regression algorithms. Consequently, the MARS algorithm can help breeders achieve enhanced outcomes within their populations. Delialioglu et al. (2023) conducted a study to predict live weight (LW) of Polatlı sheep (Ile de France × Akkaraman (G1)) considering some body measurements. The study indicated that the CART and MARS algorithms could be reliably used in morphological characterisation studies to determine indirect criteria and create elite herds regarding live weight. Özen et al. (2024) conducted a study to predict live weight using specific morphological traits, including sex, birth weight (BW), WH, CD, BL, CW, RH and CC in Akkaraman sheep. The RF, Ridge and LASSO models demonstrated optimal performance in that study.

Zhang et al. (2020) performed a case study to compare the performance of XGBoost, MARS, Artificial Neural Networks (ANN) and SVM in predicting the maximum lateral wall displacement caused by excavation and concluded that the XGBoost model yielded superior results.

## 5 Conclusion

This study has demonstrated the potential of ML algorithms in predicting weaning weight using biometric measurements before weaning age. The GBoost and XGBoost algorithms demonstrated better outcomes in terms of accuracy and generalization ability compared to other models. Researchers and livestock managers can use ML models such as GBoost and XGBoost to predict important parameters such as weaning weight in advance. This predictive capability increases to facilitate resource optimization and productivity improvements, reducing costs, which may help to achieve more economical and sustainable farming.

## Author Contributions

**Mehmet Eroğlu**: conceptualization, methodology, software, data curation, formal analysis, visualization, writing – original draft, writing – review and editing, investigation, validation. **Ali Osman Turgut**: conceptualization, data curation, formal analysis, visualization, investigation. **Mürsel Küçük**: formal analysis, data curation, validation. **Muhammed Furkan Önen**: data curation, formal analysis, investigation.

## Ethics Statement

This study was approved by the Siirt University Local Ethics Committee for Animal Experiments (Decision No: 2025‐02‐05).

## Conflicts of Interest

The authors declare no conflicts of interest.

## Data Availability

The data presented in this study are available on request from the corresponding author.
